# Implemented Interventions at the Naef K. Basile Cancer Institute to Protect Patients and Medical Personnel From COVID Infections: Effectiveness and Patient Satisfaction

**DOI:** 10.3389/fonc.2021.685107

**Published:** 2021-06-10

**Authors:** Jean El Cheikh, Samantha El Warrak, Nohra Ghaoui, Farouk Al Chami, Maya Shahbaz, Sarah Chehayeb, Nagi Saghir, Ali Bazarbachi, Ali Taher

**Affiliations:** ^1^ Bone Marrow Transplantation Program, Department of Internal Medicine, American University of Beirut Medical Center, Beirut, Lebanon; ^2^ Cancer Research Institute, Department of Internal medicine, Hematology/Oncology Division, American University of Beirut Medical Center, Beirut, Lebanon; ^3^ Faculty of Sciences, Lebanese University, Beirut, Lebanon; ^4^ Department of Nursing, Naef. K. Basile Cancer Institute, American University of Beirut Medical Center, Beirut, Lebanon; ^5^ Division of Hematology/Oncology, Department of Internal Medicine, American University of Beirut Medical Center, Beirut, Lebanon

**Keywords:** COVID-19, NKBCI, oncology, AUBMC, interventions, satisfaction

## Abstract

**Background:**

The Coronavirus Disease 2019 (COVID-19) was declared a pandemic by WHO in March 2020. The first case of COVID-19 was identified in Lebanon on the 21^st^ of February 2020, amid a national economic crisis. As the numbers of cases increased, ICU admissions and mortality rose, which led hospitals across Lebanon to take certain safety measures to contain the virus. The Naef K. Basile Cancer Institute (NKBCI) at the American University of Beirut Medical Center handles oncology outpatient visits and outpatient treatment protocol infusions. The aim of this study is to evaluate the efficacy of the safety measures put forth by the NKBCI early in the pandemic.

**Methods:**

Oncology patients are amongst the immunosuppressed population, who are at greatest risk of contracting COVID-19 and consequently suffering its complications. In this manuscript, we evaluated the precautionary measures implemented at the NKBCI of AUBMC from March 1^st^ to May 31^st^ of 2020, by surveying oncology patients on the telephone who had live and virtual appointments in both the oncology outpatient clinics and infusion unit. We conducted a prospective study of 670 oncology patients who had appointments at the NKBCI during this period and used their answers to draw responses about patient satisfaction towards those safety measures.

**Results:**

Our results involved 387 responses of oncology patients who visited the NKBCI during the period of March 1^st^ to May 31^st^ of 2020. 99% of our respondents gave a rating of good to excellent with these new measures. The option of online consultation was given to 35% in the hematology group compared to 19% in those with solid tumors (p=0.001). From the total, 15% of patients opted for the telemedicine experience as a new implemented strategy to provide patient-centered medical care. Of this group of patients, 22% faced problems with connectivity and 19% faced problems with online payment.

**Conclusion:**

NKBCI was competent in following the WHO guidelines in protecting the oncology patient population. Feedback collected from the surveys will be taken into account by the committee of the NKBCI to develop new safety measures that can better control viral spread while providing patient-centered medical care.

## Introduction

On February 21, 2020, the first case of coronavirus disease 2019 (COVID-19) was identified in Lebanon. This occurred amid a backdrop of political and economic turmoil that began in October 2019, when a banking crisis and a civil uprising pushed the country into an economic crisis (1). By mid-March, when the infection count reached 99 cases, the government declared “public mobilization,” issued stay-at-home orders, and closed the borders, with full lockdown of nonessential services (1). [*Graph demonstrating the prevalence of COVID-19 in Lebanon during the period of early outbreak of February – June illustrated in annex* (2)].

The Coronavirus Disease 2019 (COVID-19) was first recognized in December 2019 in Wuhan, China after reported cases of clusters of viral pneumonia (3). This respiratory virus causes a mild respiratory illness in 81% of affected patients, whereas 14% develop a severe illness requiring hospitalization with supplemental oxygen, and 5% progress to respiratory failure with multi-organ dysfunction (3–6).

Elderly patients, and those with comorbidities have been reported to be at a higher risk of mortality and morbidity once infected (7). Oncology patients, who are more often than not, immunosuppressed as a result of treatment from their primary disease, are at a greater risk of developing severe complications to the COVID-19 infection, with a 4-7 (average 5.6%) proposed case fatality rate (7). Cancer patients were suggested to be at a 3.5 times higher risk of severe COVID-19 disease compared to the general population (7, 8). The receipt of chemotherapy or cancer surgery was shown to be a risk factor for ICU admissions, intensive ventilation and death in COVID-19 patients (8). However, many oncology patients are postponing their clinic visits and treatment sessions due to fear of contracting the virus in overcrowded areas of the hospitals, which may hinder their care and treatment plans (9, 10).

In this manuscript, we conducted a study to address the preparation of the American University of Beirut Medical Center Oncology clinics and infusion unit, to face the COVID- 19 pandemic during the first three months of the outbreak. The Naef K. Basile Cancer Institute (NKBCI) is part of the American University of Beirut Medical Center (AUBMC). The institute handles oncology outpatient visits including outpatient chemotherapy and other regular treatment protocol infusions. AUBMC, and in turn NKBCI, are centers of care to multiple patients residing inside and outside of Lebanon.

The issuance of temporary restrictions on travel within and between countries to limit the spread of the virus has affected cancer patients seeking essential medical care. The aforementioned has blurred the lines between two healthcare necessities, protecting vulnerable cancer patients from COVID-19 and providing medical help against their disease. The NKBCI had taken additional COVID-19 response measures, along with those already implemented by AUBMC, to precisely cater to the needs of vulnerable cancer patients.

The aim of this study is to assess the measures implemented by the Hematology and Oncology Division at AUBMC, a tertiary care center in Lebanon against the challenges posed by the COVID-19 pandemic, and to incorporate the feedback of patients into the future development of these measures to better contain the virus. These measures and modifications include but are not limited to clinic interventions, remote work, patient-centered modifications, staff operations, public communication and cancer funds.

Modifications in clinic interventions include screening patients regarding recent travel and interaction with a COVID-19 infected individual ahead of their appointments, deferring appointments of patients who had recently traveled or were in contact with a COVID-19 patient and limiting entry into ACC (AUBMC Halim and Aida Daniel Academic and Clinical Center) to a single door with temperature checkpoints. Modifications in remote work include providing online consultations through WEBEX as an alternative to Hematology and Oncology clinic visits and providing physicians with remote access to hold meetings through WEBEX. Multidisciplinary tumor boards are being held in the same manner.

Patient-centered modifications include designating a separate visitor elevator to oncology patients entering ACC, laying floor markings to ensure a minimum of 2 m distance between individuals at the clinic and infusion unit check in and checkout points, decreasing the number of allowed visitors accompanying patients and deferring non-acute autologous BMT patients.

Modifications in staff operations include reducing the number of personnel in the medical team visiting the patients, emphasizing the use of PPE, appropriate hand hygiene and disinfection (4, 5, 10) and allowing staff to work from home. Public communication include live Facebook interviews to educate oncology patients and updates on AUBMC’s website about COVID-19 precautions. Modifications in NKBCI cancer funds include supporting as many outpatients and inpatients as possible.

## Methodology

This is a prospective study conducted in the NKBCI of AUBMC on patients who visited the outpatient clinics and infusion unit during the first three months of the outbreak. IRB approval was received on July 3^rd^, 2020. The primary objective of the study includes evaluating the effectiveness of precautionary strategies implemented at NKBCI to reduce risk of COVID-19 infections amongst patients and health care workers. The secondary objective of the study includes incorporating feedback from patients to improve precautionary measures against COVID-19 in the future.

The inclusion criteria include all patients who have had an appointment at the NKBCI (clinics and infusion units) between March 1^st^ and May 31^st^ of 2020. Online and in-person appointments were included. There were no exclusion criteria. Many of these patients have chronic conditions and therefore had repeat appointments during these three months, as well as follow up visits after June.

Patients were identified from the AUBMC records of patients who have visited the NKBCI outpatient clinic and infusion unit through the HIS portal on (https://his.aub.edu.lb/). Each patient’s primary physician was contacted and informed of the study prior to approaching their patient. The primary physician or their nurse contacted the patients, introduced them to the study and got their willingness to participate, after which the research team proceeded with reaching out to the patients.

A 20-question survey was created in English and translated to Arabic and French. It was administered to identified patients after their verbal consent had been taken over the telephone. The patients were given the option to either fill the survey over the phone or online through Limesurvey based on their preferences. Those 20 questions were developed by the committee responsible for the new safety measures in NKBCI, in accordance with WHO guidelines in effectively controlling viral transmission (4, 5, 11). Co-authors piloted the design and checked the feasibility and validity of the questions.

The surveys were conducted over the telephone, in a quiet and private environment in the NKBCI Research Office, with a single research member involved in the phone call. These surveys were conducted by one postdoctoral research fellow at the NKBCI, one nurse at the NKBCI, one volunteering doctor at AUBMC and one volunteering student. Individual informed consent was obtained during the period of July 20^th^, 2020 to September 18^th^, 2020. Answers were collected on a standardized excel sheet that comprised the survey questionnaire. *[Survey Questionnaire (20 questions) illustrated in annex].*

### Ethical Considerations

The Institutional Review Board at the American University of Beirut approved the study. The participants provided oral and written consent to participate in the study. The study does not involve any diagnostic or therapeutic intervention and there was no risk of harm to the patients. When consenting patients, the research assistant explained to them that study participation was merely for observational purposes and there was no influence on their future management in the hospital.

### Statistical Analysis

Following the completion of the surveys on all the respondents, the information was collected, and a simple descriptive analysis was performed. This was done using proportions and percentages for categorical variables and means and medians for continuous variables.

Numerical variables were summarized by their median, mean, standard deviation and range. Questionnaires variables were binary and restricted to yes/no answers; these categorical variables were described by counts and relative frequencies.

Crosstabs in the form of 2x2 tables were plotted to identify correlations of all variables collected in the questionnaire to be compared by visit time, gender, Hematology Patient and residence. The chi square test was referred to for correlations between categorical variables; alternatively, Fischer exact test was used when tables had cells with low counts.

Analysis was performed using SPSS software IBM v.25; a p value < 0.05 was considered statistically significant in all analyses.

## Results

**Figure 1:**
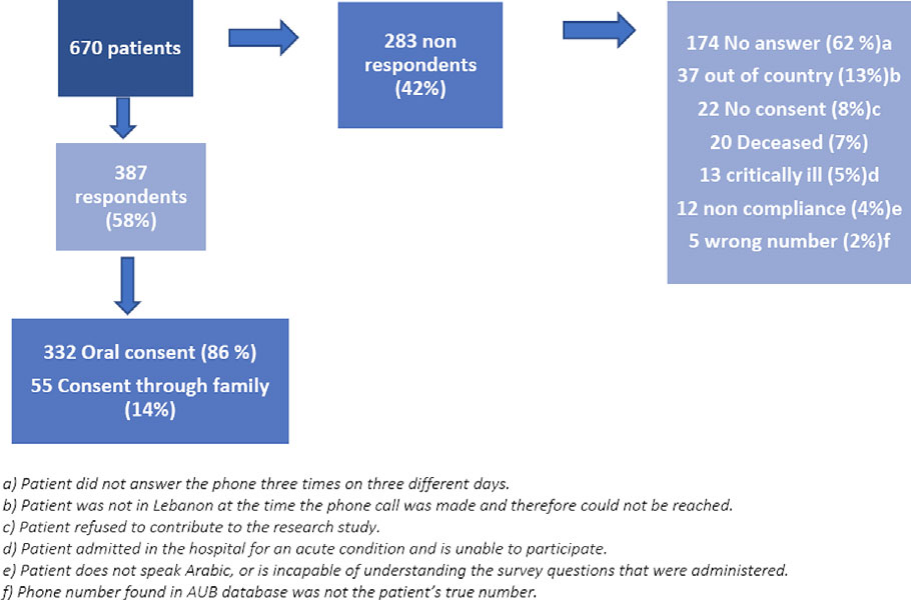
Summary of methods.

A total of 670 patients who had live and online appointments at the NKBCI (clinics and infusion unit) between March 1st and May 31st, 2020, were contacted; however, only a total of 387 responses were received (**Figure 1**). *[Patient characteristics illustrated in* **Table 1** *].*

**Table 1:** Patient characteristics.

	Total n = (%) 670	Respondents n = (%) 387	Non respondents n = (%) 283
**Sex**			
Males	250 (37.3)	137 (35.4)	113 (40)
Females	420 (62.7)	250 (64.5)	170 (60)
**Age**			
<45	177 (26.4)	114 (29.5)	63 (22.2)
45-65	305 (45.5)	167 (43.2)	138 (48.8)
>65	188 (28.1)	106 (27.4)	82 (30)
**Residence**			
Beirut	257 (38.4)	150 (38.8)	107 (37.8)
Mount Lebanon	184 (17.6)	109 (28.2)	75 (26.5)
South	65 (9.7)	36 (9.3)	29 (10.2)
Outside Lebanon	61 (9.1)	8 (2)	53 (18.7)
North	43 (6.4)	36 (9.3)	7 (2.5)
Baalbek	18 (2.7)	10 (2.6)	8 (2.8)
Bekaa	17 (2.5)	10 (2.6)	4 (1.4)
Akkar	13 (1.9)	13 (3.4)	-
Nabatiyeh	12 (1.8)	12 (3.1)	-
**Disease type**			
Solid tumors	438 (65.4)	244 (63)	194 (68.6)
Malignant Hematology	160 (23.9)	100 (25.8)	60 (21.2)
Benign Hematology	72 (10.8)	43 (11.1)	29 (10.2)

Most respondents had visited the NKBCI during March (195, 50%); Lebanon’s first nation-wide lockdown was implemented during that same month in light of increasing COVID-19 infections. The increased numbers seen in March were prior to the strict precautionary regulations implemented by both the hospital and the country, when patients and staff were less concerned about the virus. *[Prevalence of visitors across March, April, May, during the early outbreak illustrated in annex].*

Most patients’ ages ranged between 45 and 65 years of age (167, 43%) with a mean age of 55 years. *[Distribution of age ranges across respondents illustrated in annex].*

The higher numbers seen in March can be attributed to the less strict measures taken prior to the peak of the pandemic, and the less vigilance amongst patients and health care workers. Lebanon’s first nation-wide lockdown was implemented during that same month in light of increasing COVID-19 infections. The higher ratio of women to men can be attributed to the greater prevalence of breast cancer amongst oncology diagnoses, compared to other malignancies. Also, women are more willing to participate in surveys and give feedback. *[Distribution of sex ratio amongst respondents illustrated in annex].*

**Figure 2:**
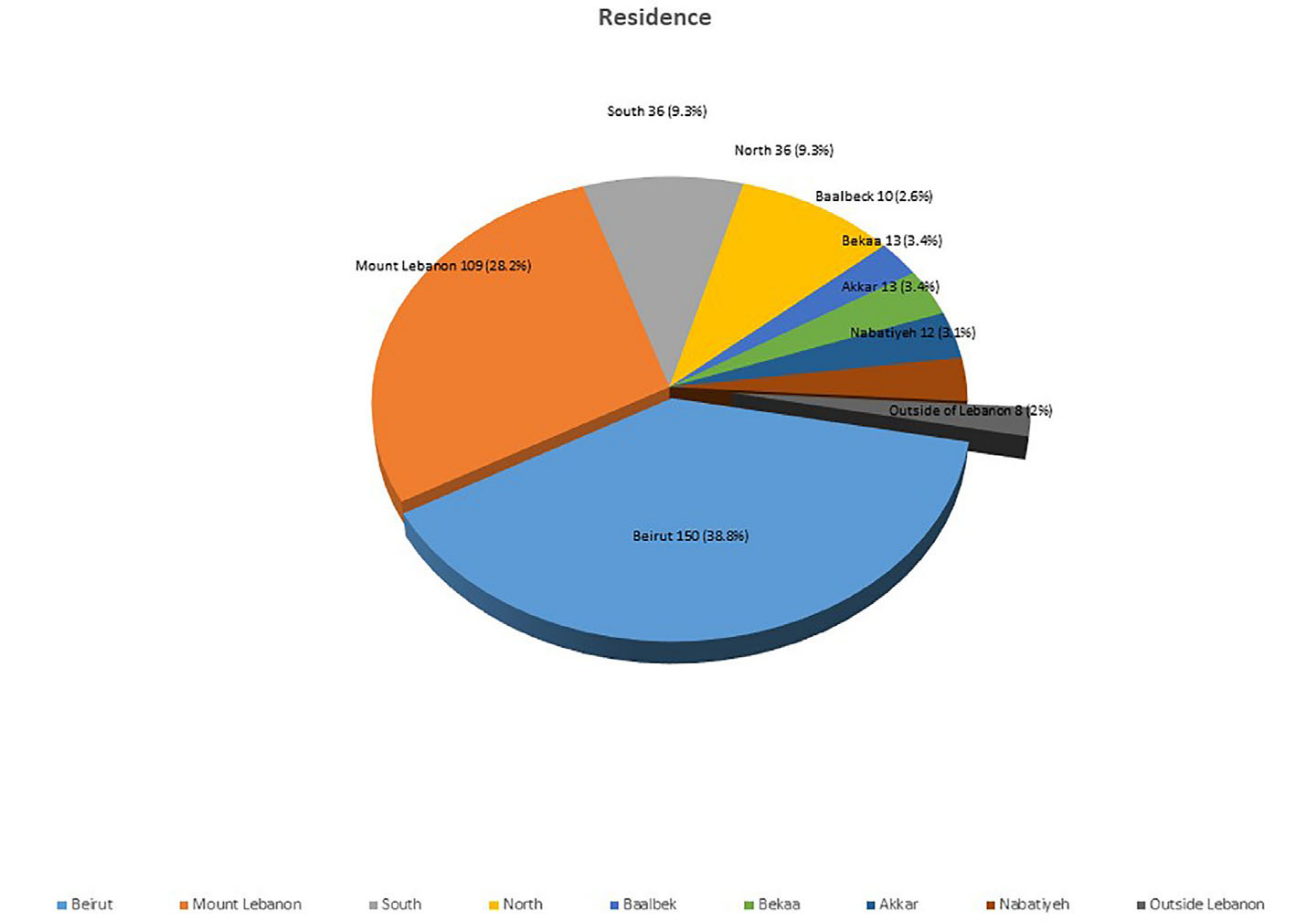
Primary residence of respondents.

370, 98% of respondents were scattered throughout Lebanon, with the remaining 8 (2%) of respondents having a primary residence outside of Lebanon. Interestingly, the majority of patients reside in Beirut (150, 39%) and Mount Lebanon (109, 28%). The small number of 2% of respondents being outside of Lebanon raises the issue of how the COVID-19 pandemic has placed travel restrictions on Lebanese living abroad and foreigners who usually travel to Lebanon to seek health care and cancer treatment. [Primary residence of respondents illustrated in **Figure 2**].

**Figure 3:**
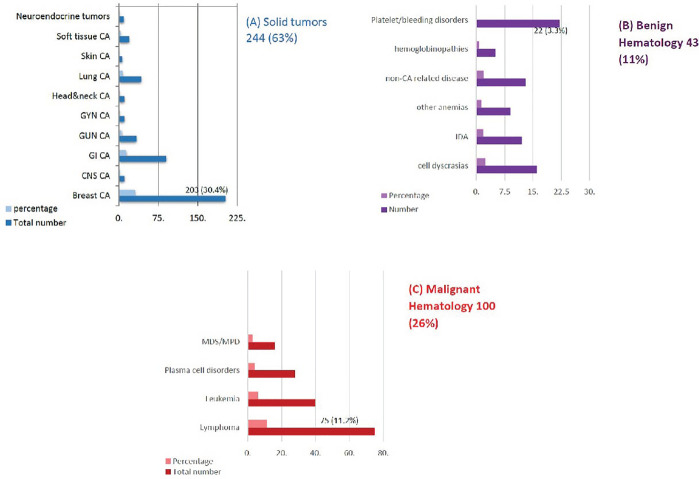
Classification of Oncology Diagnoses of respondents at NKBCI.

The patients’ respective primary diseases were identified; solid tumor patients comprised the largest patient cluster at 244 (63%) followed by benign and malignant hematology at 43 (11%) and 100 (26%), respectively. [*Classification of Oncology Diagnoses of respondents at NKBCI illustrated in* **Figure 3**].

Survey questionnaire results with values of positive and negative respondents (20 questions) illustrated in **Table 2**.

**Table 2:** Survey questionnaire results with values of positive and negative respondents (20 questions).

Pre-appointment screening out of 387 patients (7)	Yes n = (%)	No n = (%)
**1) Asked about fever/sick contacts/recent travel**	300 (77.5)	87 (22.5)
**2) Temperature taken at entrance**	377 (97.4)	10 (2.6)
**3) Reception team asked about destination**	301 (77.8)	86 (22.5)
**4) Used elevator assigned to oncology patients**	335 (86.6)	52 (13.5)
**5) Elevator occupied by ≤4 people**	320 (82.7)	67 (17.5)
**6) People committed to the assigned distance inside the elevator**	330 (85.3)	57 (12)
**7) Crowded waiting area**	81 (21)	306 (79.1)
**Role of health care worker out of 387 patients (3)**	**Yes n = (%)**	**No n = (%)**
**1) Received educational flyer about COVID-19 symptoms and precautions**	23 (6)	364 (94.1)
•**Information in the flyer clear and to the point**	23 (100)	–
•**Information in the flyer helpful**	23 (100)	–
**2) Staff interacted with wearing facemasks and gloves**	335 (86.6)	52 (13.6)
**3) Accessible sanitary supplies (hand gel, tissues, sinks)**	259 (66.9)	128 (33.3)
**Telemedicine (Telemedicine at the NKBCI during the COVID-19 Era: Providing online consultations and virtual patient care) out of 387 patients (9)**	**Yes n = (%)**	**No n = (%)**
**1) Given the option of an online CS by the operator**	95 (24.5)	292 (75.5)
**2) Opted for the online appointment**	59 (15.2)	328 (84.5)
**3) Used application by oneself without the help of another (out of the 59)**	23 (39)	36 (61)
**4) Faced problems with connectivity (out of the 59)**	13 (22)	46 (78)
**5) Faced problem with online payment (out of the 59)**	11 (18.7)	48 (81.4)
**6) Felt communicating disease status with physician and medical team changed after starting online appointments (out of the 59)**	8 (13.6)	51 (86.4)
**7) Seen: 36 in total**		
**Before the appointment**	29 (72.2)	–
**On time**	5 (13.9)	–
**Just after the appointment**	1 (2.7)	–
**8) Time devoted to the online appointment:**		
**Adequate**	30 (83.3)	–
**Inadequate**	6 (16.7)	–
**9) Knowledge, care and attention received from provider through Webex:**		
**Excellent**	14 (38.9)	–
**Good**	19 (52.8)	–
**Satisfactory**	3 (8.3)	–
**Poor**	0	–
**Patient overall satisfaction (1) **	**Yes n = (%)**	
**Overall rating of new measures against COVID-19**		
•**Excellent**	230 (59.4)	
•**Good**	131 (33.9)	
•**Satisfactory**	13 (3.4)	
•**Poor**	4 (1)	

The majority of respondents reported being screened for travel history, fever or sick contacts prior to their appointments (300, 78%). An overwhelming majority noted passing through a temperature check before entering the NKBCI (377, 97%). Most patients used the elevator reserved for oncology patients (335, 86.6%), and noted a successful implementation of social distancing in that relatively small space. A relatively significant proportion mentioned overcrowding in waiting areas (81, 21%). A few received a COVID-19 educational pamphlet (23, 6%), 100% of whom found the information clear and helpful.

52, 14% stated that staff was not wearing facemasks and gloves, and close to 130, 34% denied the presence of accessible sanitary supplies including hand sanitizers, tissues, sinks or soap. A quarter of respondents was offered the option of an online consultation (95, 25%) and close to two thirds of those patients chose that option (59, 15%). Of those respondents, half were comfortable using the application by themselves (23, 39%), but close to one-third found trouble with connectivity (13, 22%) and payment (11, 19%) and noted a negative change in communicating their disease with their physician (8, 14%). [*Demonstration of positive and negative feedback by respondents illustrated in annex*].

Males reported better pre-appointment measures compared to females, with 98% in temperature checking, 80% in being asked about their destination (compared to 95% and 76% respectively in females). Females reported greater use of specified oncology elevators (87% compared to 86% in males) and greater waiting times (23% compared to 18% in males). There was no statistical difference in relation to sex ratio and pre-appointment measures. Females reported that they had a harder time accessing sanitary supplies compared to males (63% vs 74% in males).

Females in this study were less likely to opt for the online appointment (at 42% compared to 50% in males). This could be attributed to the vast majority of breast cancer patients at NKBCI that need physical examinations. The greater numbers seen with female dissatisfaction compared to males could be due to the greater willingness of females to give detailed feedback to improve measures on the phone, the fact that most females bring along with them hand gel and tissues and therefore are less vigilant to sanitary supplies around them and that most females would prefer physical encounters rather than online consultations in the category of breast cancer population.

**Figure 4:**
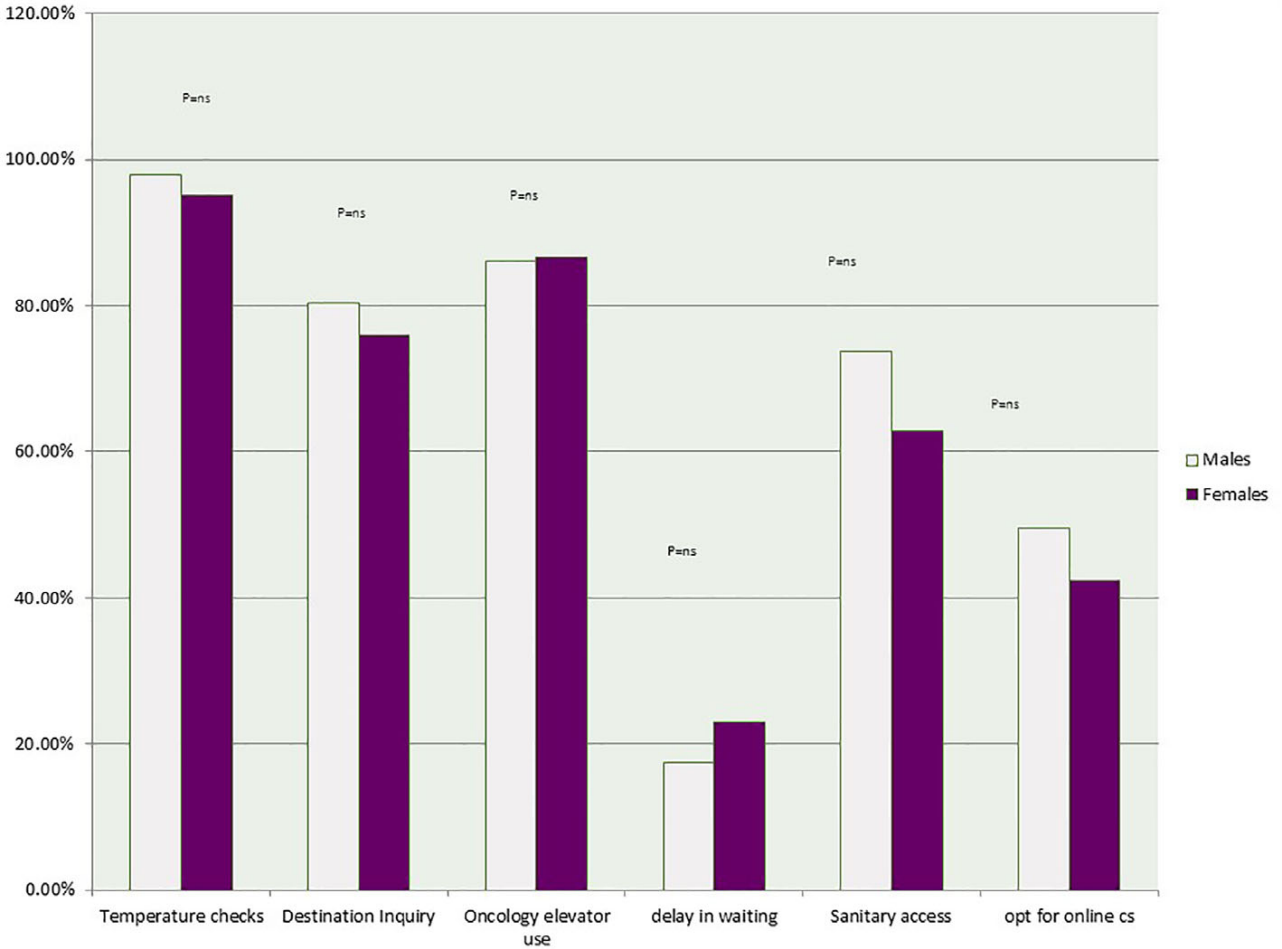
Demonstration of precautionary strategies implemented by NKBCI as per sex difference.

[Demonstration of precautionary strategies implemented by NKBCI as per sex difference illustrated in **Figure 4**].

## Discussion

As of July, Lebanon has faced an exponential increase in COVID-19 positive cases. There have been approximately 85,000 cases and 670 deaths in the first few months of the pandemic (12). Interpretation of the data yielded results that can be used to evaluate the efficacy of the new safety measures at AUBMC. This study focused on the Naef K. Basile Cancer Institute at AUBMC, which provides medical care to Hematology and Oncology patients. Given the vulnerability of the oncology patient population, extra measures had been made effective at the NKBCI to ensure patient safety and to avoid COVID-19 infections as well as the fatal complication sequelae that can arise. Though we have no direct measure of the effectiveness of these measures on our population, there is an overall sense of adequate compliance. None of the patients was infected with COVID-19 at the time of the survey. 2 patients (0.5%) had tested positive for COVID-19 during the early outbreak of the virus and not when the study was conducted, with only mild symptoms and no hospital admissions. One patient was a 54-year-old female with Sigmoid Cancer and the other was a 45-year-old female with acquired eosinophilia. The fewer COVID-19 cases seen in oncology clinics in the beginning of the pandemic are due to the fact that this group of patients is at a greater fear of contracting the virus and therefore follows stricter precautionary measures overall compared to the general population (7).

Our results show that the COVID-19 safety strategies were not strongly implemented in the beginning of the pandemic wave in Lebanon (early March), where patients reported lack of supervised elevators and temperature checks at the entrance. These strategies grew stronger towards the end of March. 5 patients (1%) however, personally suggested that strategies were much stronger early in the outbreak. This could be attributed to the leniency of the protective measures towards the summer and the lifting of the national lockdown in July (1). 37% of patients reported better measures in April compared to 29% in March (p=0.003).

Precautionary measures were not implemented in the valet parking areas, where patients complained about the crowded groups posing a risk of COVID-19 transmission. 6 patients (2%) were dissatisfied with the parking service, which is the first point of contact with the hospital premises. There was no statistical difference between patient satisfaction to the measures at NKBCI and their gender or place of primary residence. 35% of patients in the hematology group compared to 19% in those with solid tumors were given the option of an online consultation (p=0.001), likely due to the higher risk of myelosuppression associated with hematology patients.

The majority of responses showed an adherence to social distancing guidelines on hospital premises and the equally important temperature checks. Overcrowding in waiting rooms seems to be a persistent issue. Fewer staff that are currently working during the pandemic have allowed for greater delay times at waiting desks. A future strategy to mitigate this issue would be stricter visitor policies, or on-boarding more staff with effective social distancing measures in order to reduce waiting times (13). This increase in waiting time during the outbreak has shown to be more prominent than the pre-pandemic era.

COVID-19 educational pamphlets should have been administered at a larger scale as evidenced by the small number of patients who was given information booklets; however, this is accompanied by the risk of physical contact with surfaces that could harbor the virus. This topic can be addressed by introducing electronic means of spreading information about the virus, rather than data in hard copy that can allow for viral transmission along surfaces. Alternating seats are amongst the new social distancing measures taken by hospitals. Although this can help reduce the risk of transmission of the virus, the elderly and the cachectic population (common amongst oncology patients) might be forced to remain standing during waiting hours. There must be more seats available to accommodate all the patients that will be seen in the clinic, whilst still practicing effective social distancing (14).

Telemedicine (TM) has become the platform of patient-centered care in the pandemic. Telemedicine permits mildly ill patients to get the supportive care they need while minimizing their exposure to other acutely ill patients (15). The implementation of the new EPIC EHR system at AUBMC in November 2018 made way to new opportunities in remote patient-centered care and telemedicine. Only 59 (15%) reported to have used telemedicine during the pandemic. 14 patients (24%) who opted for virtual appointments would like to continue online consultations in a COVID free era. Some of these patients gave reasons such as distant residence and a long drive to get to the hospital, delayed waiting times in the emergency department, delay on the phone to schedule a live appointment, etc. Some would opt for an online appointment if they had the means to use technology at home (lack of computer for example). Others would not opt for an online appointment because of the need to be examined personally by their primary physician (particularly breast cancer patients).

Unfortunately for the economic crisis in Beirut, 11 patients (19%) were unable to pay for the online appointment *via* the Internet and could only pay in cash the next time they would visit the hospital. This raises the issue about putting such patients’ lives at risk by bringing them personally to the hospital to make a payment, when the primary purpose of telemedicine is to limit their exposure to COVID-19. The Internet connection in Lebanon is also problematic. 13 patients (22%) complained about the inability to continue with their online consultation due to connection problems, and as such would choose to see the doctor live for their next appointment.

Telemedicine can pose the risk of ineffective history taking. From our respondents who underwent an online consultation, 3 patients (5%) did not get asked about fever, sick contacts or recent travel during initial history taking because they did not need to come to the hospital. Those patients are predisposed to a significant amount of immunosuppression and therefore, it is important to thoroughly screen for viral symptoms, even if they have virtual appointments and do not need to physically come to the hospital. For patients who only undergo continuous chemotherapy and immunotherapy in the infusion unit of NKBCI, online consultations are inapplicable to them, and therefore they could not contribute to this part of the study.

Bone marrow transplant centers have been particularly affected during the pandemic. Non-urgent auto transplants were postponed during the COVID-19 era, however, inpatient transplant care during the pandemic was not addressed in this study. Patients undergoing transplants were not surveyed due to their incapability to participate in their critical conditions. To protect individuals from SARS-CoV-2 infections, it can only be recommended that maximum emphasis should be placed on personal protection (16, 17). Recommendations for donor screening and detailed medical history (including travel history, exposure to COVID-19 etc.) should be followed (16–20).

The data collected from the patients can be used to draw an effective action plan. The feedback received from patients who had appointments at the NKBCI during the beginning of the pandemic is necessary to help implement new effective strategies in the future control of COVID-19. The online portal for patients must be improved so as not to face difficulties with payment or connection during virtual consultations. More stuff must be hired to control crowded waiting areas while still implementing social distancing and safety measures. Elevators should be kept supervised throughout the pandemic and not only during national lockdown measures. There should be a greater supply of sanitary supplies throughout the hospital premises and not only inside clinics. The number of seats in waiting areas can be increased to allow the frail population to rest while waiting, all whilst still practicing effective social distancing. There must be greater supervision in the valet parking areas. The frequency of use of electronic means of communication must be increased (information leaflets, medication prescription, laboratory tests, medical reports etc.) to limit the transmission of the virus *via* surfaces. A higher proportion of successful, problem-free virtual encounters should be the goal in the near future. A history of travel to a highly affected area is likely to become irrelevant as part of the pre-screening protocol as more areas have become affected. TM can be used for ongoing management of other chronic diseases such as asthma and immunodeficiency disorders, as individuals with these conditions are particularly susceptible to COVID-19, and medication compliance and disease optimization are important ways to mitigate severity (15).

The strengths of the study include the fact that this is the first survey done in the region, at one of the top tertiary medical centers in Lebanon and the Middle East that provides elite patient care, to assess the changes made during the COVID-19 pandemic. In addition, the study was conducted in a single center, homogenous population using a big sample number of 670 oncology patients. The limitation of the study is that it solely relies on a telephone based source. Another limitation is that a big number of 263 did not respond (excluding the deceased). The study was done over a short duration of the first three months of the outbreak, therefore; only limited information could be obtained. The study can be extended over 12 months to incorporate a bigger sample of patients, and to study the changes in precautionary strategies as the pandemic advances.

## Conclusion

The above recommendations will be taken into account by the committee of the NKBCI to develop new safety measures that can better control viral spread amongst the oncology patient population, while maintaining best patience care in the hospital. The study can be extended over the next 12 months to evaluate the efficacy of these new precautionary strategies in accordance with the change in COVID-19 behavior across the year.

## Data Availability Statement

The original contributions presented in the study are included in the article/**Supplementary Material**. Further inquiries can be directed to the corresponding author.

## Ethics Statement

The Institutional Review Board at the American University of Beirut approved the study. The participants provided oral and written consent to participate in the study.

## Author Contributions

JC created the concept of the study, wrote, revised and edited the manuscript. SW was involved in data collection, statistical analysis and writing the manuscript. NG, FC, MS, and SC contributed to data collection. NS, AB, and AT read, revised and commented on the manuscript, and approved the submitted version. All authors contributed to the article and approved the submitted version.

## Supplementary Material

The Supplementary Material for this article can be found online at: https://www.frontiersin.org/articles/10.3389/fonc.2021.685107/full#supplementary-material

## Conflict of Interest

The authors declare that the research was conducted in the absence of any commercial or financial relationships that could be construed as a potential conflict of interest.
