# Supplementary material for: Implemented Interventions at the Naef K. Basile Cancer Institute to Protect Patients and Medical Personnel From COVID Infections: Effectiveness and Patient Satisfaction
Source: Front Oncol. 2021 Jun 10;11:685107. doi: 10.3389/fonc.2021.685107 (PMC8237752; doi:10.3389/fonc.2021.685107)
Supplement: Supplementary file 1 [file Table_1.docx]

Data collection sheet:

| Name |
| --- |
| MRN |
| Oral Consent |
| Date of visit |
| Clinic/infusion unit |
| When you scheduled your appointment at the oncology department, did the operator ask you about any of the following (Fever, sick contacts or recent travel history) yes/no |
| Was your temperature taken at the hospital’s entrance? Yes/no |
| Did the reception team ask you about your destination? Yes/no |
| Did you use the elevator assigned for oncology patients? Yes/no |
| Was the elevator occupied by more than 4 people? Yes/no |
| Did the people commit to the assigned distance inside the elevator (footsteps sign on the floor) yes/no |
| Was the waiting area crowded? Yes/no |
| Did you receive an educational flyer about Covid-19 symptoms and precautions? Yes/no |
| Did you find the information in the flyer clear and to the point? Yes/no |
| Did you find the information in the flyer helpful? Yes/no |
| Were the staff that you interacted with wearing facemasks and gloves? 1)Yes, all staff were protected 2) Some yes, others no 3) No staff was wearing any protection |
| Were the sanitary supplies accessible to you (handgel, sinks, tissues...etc)? Yes/no |
| Were you given the option of the online consultation by the operator? Yes/no |
| Did you opt for the online appointment? 1)Yes 2)No, I usually get help from someone else 3) Not applicable |
| Do you use the application by yourself? Yes, no, na |
| Did you face any problem with connectivity? Yes, no, na |
| Did you face any problem with online payment method? Yes, no, na |
| For your scheduled appointment, were you seen 1)Before your appointment 2)On time 3)Just after 4)Long after 5)I was late 6) N/A |
| Do you feel that communicating your disease status with your physician and the medical team has changed after you started the online appointments? Yes, no, na |
| Time devoted to the online appointment:1)Adequate 2)Not Adequate 3)N/A |
| Please rate the knowledge, care and attention you received from your provider through Webex? 1)Excellent 2)Good 3)Satisfactory 4)Poor 5)N/A |
| Overall, how do you feel about the new measures?1)Excellent 2) Good 3)Satisfactory 4) Poor |
| any suggestions or complaints? |
